# The War of Secession (1861-5)

**Published:** 1899-10

**Authors:** 


### Abstracts and Selections.

### The War of Secession (1861-5).—Historical Data.

[The following is taken from the report of Surgeon-General C. H. Tebault, United Confederate Veterans, read at the Charleston, S. C., Confederate reunion, June, 1899, and published in *Gaillard's Medical Journal*, August, 1899.—ED.]

"Let me here briefly and tersely recite a few historic facts from official data in my possession, of interest to stimulate our further research: Of the thirty-four States and Territories, only eleven seceded. In these eleven States the men of military age—from eighteen to forty-five years—numbered 1,064,193, inclusive of lame, halt and blind, etc. On the Union side the same class numbered 4,559,872, over four to one, without estimating the constant accessions from the world at large, augmenting monthly the Union side.

"The United States, in enlisted men, numbered 2,865,028, against not exceeding 600,000 on the side of the Southern Confederacy.

"With the States of Kentucky, Missouri, Maryland, West Virginia, Tennessee, and the remainder of the Southern States, the remarkable fact presents that the South itself—the slave States—gave exceeding 300,000 men to the Union side, more than half as many soldiers as comprised the entire Confederate army. These above facts, derived from the war records, show that there were four armies in the field, each one of which was as large as the entire Confederate army, without including the more than 300,000 contingent from the South.

"In numbers the Federal loss was 67,058 killed, and 43,012 died of wounds; total, 110,070. Of the Confederates, the like total was 74,524. The Confederates had 53,773 killed outright, and 194,026 wounded on the field of battle. More than one-third of the 600,000 Confederates were, therefore, confided to the Confederate surgeons for battle wounds. For the nineteen months—January, 1862, to July, 1863, inclusive—over 1,000,000 cases of wounds and sickness were entered upon the Confederate field reports, and over 400,000 cases of wounds upon the hospital reports. It is estimated that each of the 600,000 Confederates were on an average disabled for greater or less periods by wounds and sickness about six times during the war. The heroic, untiring, important part thus borne by the skillful Confederate surgeons in maintaining in the field an effective army of unexampled Confederate soldiers must challenge particular attention.

"The destruction by fire of the medical and surgical records of the Confederate States deposited in the Surgeon-General's office in Richmond, Va., in April, 1865, renders the roster of the medical corps somewhat imperfect, hence the need of concerted action on the part of the survivors to bridge this hiatus. The official list of the paroled officers and men of the Army of Northern Virginia surrendered by General R. E. Lee, April 9, 1865, furnished 310 surgeons and assistant surgeons. In my first report, presented at the Richmond reunion, I showed that the medical roster for the Army of Tennessee has been preserved in duplicate. I shall offer in a more detailed report data to prove indisputably important facts relating to the prisoners of war upon both sides, with the purpose of establishing the death rate responsibility in the premises. It will suffice to mention here that the report of Mr. Stanton, as Secretary of War, on the 19th of July, 1866, exhibits the fact that of the Federal prisoners in Confederate hands during the war only 22,570 died, while of the Confederate prisoners in Federal hands 26,436 died. This report does not set forth the exact number of prisoners held by each side, respectively.

"These facts were given more in detail in a subsequent report by Surgeon-General Barnes, of the United States Army.

"That the whole number of Federal prisoners captured by the Confederates and held in Southern prisons from first to last during the war was, in round numbers, 270,000, while the whole number of Confederates captured and held in prisons by the Federals was, in like round numbers, only 220,000. From these two reports it appears that, with 50,000 more prisoners in Southern stockades or other modes of confinement, the deaths were nearly 4000 less! According to these figures, the per centum of Federal deaths in Southern prisons was under 9, while the per centum of Confederate deaths in Northern prisons was over 12. These mortuary statistics are of no small weight in determining on which side there was the most neglect, cruelty and inhumanity, proclaiming as they do a loss by death of more than three per cent. of Confederates over Federals in prisons, while the Federals had an unstinted command of everything.

"There is in my keeping unchallenged evidence to demonstrate that the refusal to exchange prisoners was not due to the Confederate government."

